# New approach to busulfan dosing in infants and children based on a population pharmacokinetic analysis

**DOI:** 10.1007/s00280-025-04757-w

**Published:** 2025-02-11

**Authors:** Frank M. Balis, Elizabeth Rieger, Nancy J. Bunin, JoAnn Gardiner, Leslie M. Shaw, Timothy S. Olson, Michael C. Milone

**Affiliations:** 1 Division of Oncology, Children’s Hospital of Philadelphia and University of Pennsylvania, Perelman School of Medicine, Philadelphia, PA 19104 USA; 2 Department of Pathology and Laboratory Medicine, University of Pennsylvania, Perelman School of Medicine, Philadelphia, PA 19104 USA; 3 Children’s Hospital of Philadelphia, 3401 Civic Center Blvd, CTRB4024, Philadelphia, PA 19104 USA

**Keywords:** Busulfan, Body surface area, Body weight, Therapeutic drug monitoring, Stem cell transplantation, Dosing table

## Abstract

**Purpose:**

Apply population pharmacokinetic modeling to a single institution busulfan therapeutic drug monitoring (TDM) data set from infants and children to refine dosing methods.

**Methods:**

One-compartment pharmacokinetic model was fit to busulfan TDM data from 328 infants and children with malignant and non-malignant diseases treated with busulfan-containing transplant conditioning regimens. Age-dependence of busulfan clearance scaled to body weight and body surface area (BSA) was compared, and busulfan AUC was simulated for a BSA-scaled dose of 100 mg/m^2^ combined with a BSA-banded dosing table for infants and children with a BSA < 0.5 m^2^.

**Results:**

Busulfan clearance scaled to body weight is age-dependent. Clearance in children ≤ 3 years (0.234 L/[h•kg]) is higher than the typical value for the population, (0.205 L/[h•kg]), and 48% of children < 5 years have subtherapeutic busulfan AUCs after the first dose. Busulfan clearance scaled to BSA (typical value, 5.47 L/[h•m^2^]) is more uniform across the pediatric age span, except for infants (≤ 1 year, 4.27 L/[h•m^2^]). Simulated busulfan AUCs with a dose of 100 mg/m^2^ for patients with a BSA ≥ 0.5 m^2^ combined with a BSA-banded dosing table for patients with a BSA < 0.5 m^2^ achieved a therapeutic AUC after the first dose in 49% more patients than body weight scaled doses.

**Conclusion:**

Our model predicts a greater proportion of children would achieve a therapeutic busulfan *AUC* after the first dose with a dose of 100 mg/m^2^/d combined with the infant dosing table for patients with a BSA < 0.5 m^2^ compared to body weight-scaled dosing.

**Supplementary Information:**

The online version contains supplementary material available at 10.1007/s00280-025-04757-w.

## Introduction

Busulfan is commonly included in conditioning regimens for hematopoietic stem cell transplants (HSCT). The starting dose is typically scaled to body weight for children, and the dose is subsequently adjusted using therapeutic drug monitoring (TDM) to achieve an area under the concentration-time curve (AUC) within a narrow therapeutic range [1]. The use of TDM has generated large pharmacokinetic data sets that have been used in population pharmacokinetic analyses to identify factors contributing to inter-patient variability and to refine dosing methods [2].

The doses of most anticancer drugs in children are scaled to body surface area (BSA) rather than body weight. This approach evolved from the practice of using BSA to scale drug doses from animal models to humans and from adults to children [3]. Drug clearances scaled to BSA are more uniform across the pediatric age range than clearances scaled to body weight, and as a result, drug exposures (*AUCs*) are also more uniform when drug doses are scaled to BSA [4]. However, BSA over-predicts clearance in infants, and extrapolating doses of anticancer drugs to infants based on BSA resulted in excessive toxicity [5, 6]. Conversely, clearances normalized to body weight are higher in young children, and higher doses per kg may be required to achieve desired drug exposures [7]. Neither of these dose scaling methods is satisfactory for all age groups [8–10].

A wide variety of dose modification methods were developed for conventional doses of anticancer drugs in infants to address the enhanced toxicity observed with BSA-scaled dosing [4]. Recently, a unified method using BSA-banded infant dosing tables was developed by the Children’s Oncology Group’s (COG) Chemotherapy Standardization Task Force and subsequently implemented in new COG clinical trials [4].

We report on a population pharmacokinetic analysis of busulfan from our single institutional experience with a focus on infants and propose a new dosing approach employing BSA-scaled dosing with a BSA-banded dosing table (Table 1) for infants and young children with a BSA < 0.5 m^2^.

**Table 1:** BSA-banded busulfan infant dosing table for patients with a BSA < 0.5 m^2^. The dose in mg is selected from the table based on which BSA band the patient’s BSA falls within for each schedule. For example, and infant with a BSA of 0.33 m^2^ would receive a daily dose of 25 mg on the daily schedule

	Dosing Schedule	
BSA [m^2^]	Daily	q6h
0.2–0.24	18 mg	4.5 mg
0.25–0.29	20 mg	5 mg
0.3–0.34	25 mg	6.25 mg
0.35–0.39	32 mg	8 mg
0.4–0.44	40 mg	10 mg
0.45–0.49	50 mg	12.5 mg
≥ 0.5	100 mg/m^2^	25 mg/m^2^

## Subjects and methods

### Population

Patients with malignant (*n* = 172) and non-malignant (*n* = 156) diseases treated with hematopoietic stem cell transplant (SCT) after conditioning with a busulfan-containing regimen at the Children’s Hospital of Philadelphia between Feb 2007 and May 2023 were included in this study if they had plasma busulfan concentrations monitored after their first dose of busulfan at the University of Pennsylvania Clinical Toxicology Laboratory. This retrospective study was reviewed by Penn Institutional Review Board and qualified for an IRB review exemption.

Data elements abstracted from each subject’s medical record are listed in Supplemental Table 1. Patient age was calculated from the date of birth and the date that the first dose of busulfan was administered. Laboratory values for assessing kidney and liver function were within the reference interval for essentially all subjects and, therefore, were not included as covariates in the population pharmacokinetic analyses.

### Busulfan dosing and sampling schedule

Busulfan was administered in divided doses over 4 days in most patients. Prior to 2016, busulfan was given in 16 divided doses every 6 h (q6h) over 96 h, and from 2016 onward busulfan was administered as four daily doses, except for seven children with neuroblastoma treated with four daily busulfan doses prior to 2016 and 22 infants < 12 months of age treated on the q6h dosing schedule after January, 2016. Twenty-nine subjects received fewer doses of busulfan administered over 48 h, including 26 patients (17 with non-malignant disorders) treated with 50% of the total dose administered in 8 divided doses q6h and three patients with Fanconi anemia treated with 20% of the standard dose administered every 12 h for 4 doses.

The starting dose of busulfan was scaled to body weight. The dose was 3.2 mg/kg/dose (12.8 mg/kg/course) infused over 3 h on the daily x 4 schedule and 0.8 mg/kg/dose (12.8 mg/kg/course) infused over 2 h on the q6h x 16 schedule. Thirty-eight children who were > 10 kg and < 48 months of age received a 25% higher dose (4 mg/kg/dose) on the daily schedule. Forty-four patients who were treated on the q6h schedule received 1 mg/kg/dose, including 36 patients who were > 10 kg and < 48 months old and 5 patients who were < 3 months old. The dose was scaled to adjusted ideal body weight (AIBW) in 9 patients who were obese. The actual administered doses were used for pharmacokinetic analyses.

Six or seven plasma samples were drawn after the first infusion of busulfan on the first day, and 6 plasma samples, including a pre-infusion sample and 5 post-infusion samples, were drawn on days 2 and 3, if necessary. Supplemental Table 2 lists the sampling times for the daily and q6h dosing schedules. The actual sample times for each patient were used for pharmacokinetic analyses.

The busulfan dose was adjusted to achieve an *AUC*_*inf*_ (AUC extrapolated to infinity) between 3600 and 6000 µM•min (14.8 and 24.6 mg•h/L) assuming a linear relationship between busulfan dose and busulfan *AUC*_*inf*_. Most patients who had dose adjustments were monitored after the first busulfan infusion on day 2 and after the first busulfan infusion on day 3, if a second dose adjustment was required on day 2, to ensure that the busulfan *AUC*_*inf*_ was within the therapeutic range.

### Busulfan assay

Plasma samples were analyzed for busulfan using a gas chromatography/mass spectrometry method with selected ion monitoring as previously described [11]. Busulfan concentrations were reported as ng/mL.

### Pharmacokinetic analysis

For clinical purposes, the busulfan *AUC*_*inf*_ was estimated in real time by fitting a one-compartment model with first-order elimination to the busulfan concentration-time data using Phoenix WinNonlin, versions 7 and 8 (Certara, Princeton, NJ) in compliance with the vendor’s ‘Acceptable Use Policy.’ AUCs for all monitored doses were recalculated using the trapezoidal rule in Phoenix’s NCA module and were in close agreement (mean difference 1.7%) with the clinically reported values. We use the harmonized busulfan plasma exposure unit (mg•h/L) for reporting *AUC*_*inf*_ [12]. For population pharmacokinetic modeling, we used Phoenix NLME v.8.3 (Certara USA, Inc, Princeton, NJ). Population pharmacokinetic analyses used a one-compartment model with first-order elimination, parameterized with clearance (*CL*) and volume of distribution (*V*). This model has been used in 78% of prior population pharmacokinetic models for busulfan [2]. Supplemental Table 3 lists the 20 model fits performed. The actual dose was input as ng, ng/kg, or ng/m^2^, and the dose per kg and dose per m^2^ were calculated from each patient’s actual dose and their pre-treatment weight and BSA. A multiplicative random error model and the FOCE-ELS (First-Order Conditional Estimation, Extended Least Squares) algorithm in the Simple run mode were used for all population pharmacokinetic model fits. The Covariate Search Stepwise run mode was used to evaluate body weight and BSA as covariates for *V* and *CL*. Covariates were normalized to median values of BSA (0.81 m^2^) and body weight (20.5 kg). -2•log likelihood was the objective function used to discriminate among scenarios, and the p-value to add a covariate was 0.01 and to remove a covariate was the p-value was 0.001. Goodness of fit of models was assessed from Phoenix log plots of measured vs. predicted individual concentrations (log DV vs. log iPRED) and from conditional weighted residual plots (CWRES). A bootstrap analysis with 1,000 samples and a maximum of 10 tries was performed with the final model incorporating covariates to test robustness of the model. Individual patient parameters (*V*_*i*_ and *CL*_*i*_) were obtained from Phoenix’s Post Hoc analysis table. Individual parameters are derived from the population typical values of the parameters (*tvCL* and *tvV*), which represent the fixed effects, and the post hoc values of the random effects (*hV* and *hCL*) with the equations:

$$\:{V}_{i}=tvV\cdot\:{e}^{{\eta\:V}}\:\text{a}\text{n}\text{d}\:{CL}_{i}=tvCL\cdot\:{e}^{{\eta\:CL}}$$.

Busulfan *AUC*_*inf*_s were simulated for each patient for BSA-scaled doses of 100 mg/m^2^. For patients with a BSA < 0.5 m^2^, *AUC*_*inf*_s were also simulated using a dose taken from a BSA-banded infant dosing table (Table 1). AUCs were calculated using the equation:

$$\:{AUC}_{i}=\:\frac{Dose}{{CL}_{i}}$$

The BSA-banded infant dosing table was developed by grouping patients with a BSA < 0.5 m^2^ into bands at 0.05 m^2^ increments and estimating the dose (in mg) required to achieve a busulfan *AUC*_*inf*_ in the middle of the therapeutic range using the patients’ individual *CL*_*i*_s (*Dose*_*i*_*=* [*desired AUC*]*•CL*_*i*_) and taking the average of *Dose*_*i*_ for each BSA band.

## Results

### Patient characteristics

Three-hundred-twenty-eight patients received busulfan for conditioning prior to a HSCT and had therapeutic drug monitoring after the first dose of busulfan. Patient characteristics are provided in Table 2. Supplemental Table 4 lists the other drugs administered with busulfan in the conditioning regimens. The median age was 5.9 years on the day the first dose of busulfan was administered and 27% of the population was under 2 years of age (Supplemental Fig. 1). Three patients were over 21 years of age.

**Table 2:** Patient characteristics

Characteristic		Units
Number of patients	328	
Number of samples	3339	
Median (range) Age	5.9 (0.11 to 28.1)	years
Sex	195 males 133 females	
Median (range) Weight	20.5 (3.4 to 126)	kg
Median (range) BSA	0.81 (0.21 to 2.42)	m^2^
Dosing schedule		
q6h Daily	174 154	
Median (Range) Total Busulfan dose q6h schedule x 16 doses (*n* = 148)	277 (60–853) 14.4 (4.8–23.2) 375 (116–548)	mg mg/kg mg/m^2^
q6h schedule x 8 doses (*n* = 26)	138 (30.4–840) 6.83 (3.07–12.5) 187 (71.9–284)	mg mg/kg mg/m^2^
Daily schedule x 4 doses (*n* = 151*)	385 (78.5–1460) 13.3 (6.5–22.6) 405 (178–622)	mg mg/kg mg/m^2^
Disorder	131 Leukemia 97 Immunodeficiency 47 Hematologic (non-malignant) 36 Neuroblastoma 12 Inborn error of metabolism 5 Lymphoma	
Donor	43 Autologous 42 Haploidentical 74 Matched sibling 169 Unrelated, including cord blood	

* Three patients with Fanconi treated with reduced dose not included

One-hundred sixty-seven patients were monitored after the first dose on day 2, and 39 patients were also monitored after the first dose on day 3. One-hundred thirty-one patients had a busulfan dose increase on day 2, 41 had a dose decrease, and the dose was not adjusted in 156 (48%) patients.

### Noncompartmental Analysis

**Fig. 1:**
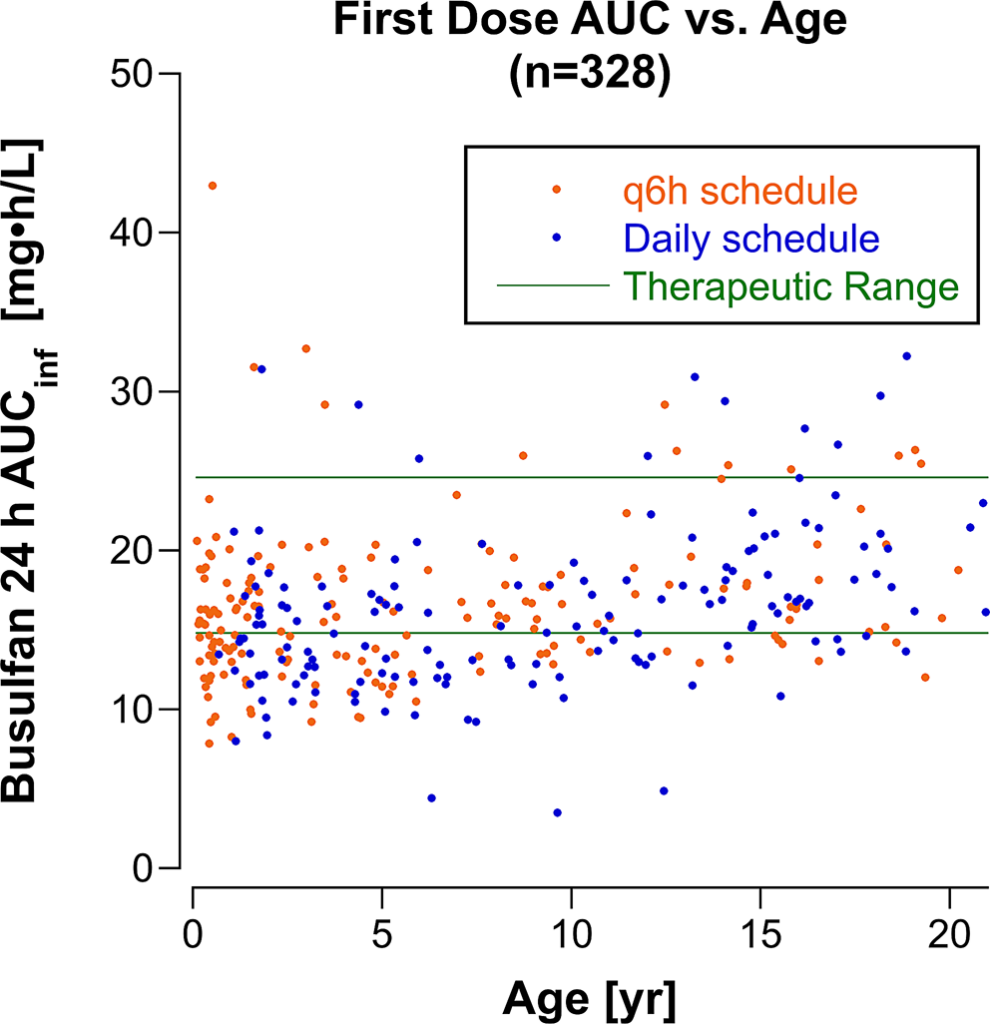
Busulfan *AUC*_*inf*_ as a function of patient age after the first dose in 328 patients. *AUC*_*inf*_ for patients on the q6h schedule was multiplied by 4 to give the *AUC*_*inf*_ achieved with 4 doses administered over 24 h (equivalent to the AUC_inf_ from the daily dosing schedule)

The mean ± SD *AUC*_*inf*_ from the NCA analysis after the first dose on the daily schedule was 16.2 ± 5.1 mg•h/mL (3946 ± 1242 µM•min) in 154 patients, and on the q6h schedule the *AUC*_*inf*_ was 4.12 ± 1.23 mg•h/mL (1004 ± 300 µM•min) in 174 patients. The *AUC*_*inf*_ was within the therapeutic range in 168 (51%) patients, below the therapeutic range in 136 (41%) patients and above the therapeutic range in 24 (7%) patients. Figure 1 shows the relationship of *AUC*_*inf*_ to patient age. Younger children were more likely to have a subtherapeutic *AUC*_*inf*_ on day 1. The total *AUC* for the 4-day dosing period (incorporating the *AUC*s measured on days 2 and 3 after dose adjustments and excluding the 29 patients who received a reduced total busulfan dose) was within the therapeutic range in 268 of 299 patients (90%), subtherapeutic in 25 (8%) patients and above the therapeutic range in 6 (2%) patients.

The mean ± SD busulfan *AUC*_*inf*_ after the first dose in 9 patients whose dose was scaled to AIBW was 17.3 ± 3.6 mg•h/L and was subtherapeutic in 2 patients.

The percent change in the day 2 *AUC*_*inf*_ from day 1 in 156 patients who required a dose adjustment was disproportionately higher by a median of 16% than would be expected based on the percent change in the busulfan dose from day 1 to day 2 (Supplemental Fig. 2). The day 3 *AUC*_*inf*_ was a median of 4.8% higher than would be expected based on the percent change in the busulfan dose from day 2 to day 3 in 39 patients.

### Population pharmacokinetic analysis

The one-compartment model adequately describes the busulfan concentration-time data across a broad range of ages and body sizes (Supplemental Fig. 3). Population typical values for busulfan *CL* and *V* for a one-compartment model for the entire population and for subsets of the youngest patients are shown in Table 3. *CL* scaled to body weight (L/[h•kg]) is higher in patients ≤ 3 years of age than the population *CL*, whereas *CL* scaled to BSA (L/[h•m^2^]) is lower in the youngest patients, especially in those ≤ 1 year of age, compared to the population *CL*.

**Table 3:** Typical values (RSE) for *CL* and *V* for a one-compartment model fit to the busulfan plasma concentration-time data from the entire population and subsets of the youngest patients. Separate model fits were performed with dose input as ng, ng/kg and ng/m^2^. The group ≤ 3 years includes the patients ≤ 2 years and patients ≤ 1 year, and the group ≤ 2 years includes the patients ≤ 1 year

		Clearance (RSE*)			Volume of distribution (RSE)		
Patients	*n*	L/h	L/(h•kg)	L/(h•m^2^)	L	L/kg	L/m^2^
All	328	4.43 (3.77)	0.205 (1.60)	5.47 (1.40)	14.5 (4.20)	0.668 (0.97)	17.8 (1.38)
≤ 3 years	108	2.12 (4.45)	0.234 (2.42)	4.95 (2.76)	6.24 (3.89)	0.685 (1.82)	14.5 (2.16)
≤ 2 years	88	1.92 (4.73)	0.228 (2.72)	4.74 (2.99)	5.70 (4.17)	0.675 (2.14)	14.0 (2.42)
≤ 1 year	46	1.46 (5.10)	0.218 (3.80)	4.27 (3.75)	4.55 (4.53)	0.679 (2.39)	13.3 (3.39)

* RSE, Relative standard error expressed as % of the parameter estimate

The busulfan *CL* in males (median, 5.65 L/[h•m^2^]) and females (median, 5.38 L/[h•m^2^]) are compared in Supplemental Fig. 4 and are not statistically significantly different.

Population pharmacokinetic parameters scaled to BSA were similar for the daily and q6h dosing schedules. The group treated on the q6h schedule were younger (median age, 4.11 year) than the group treated on the daily schedule (median age, 9.02 year). On the daily schedule (*n* = 154) the *V* was 19.6 L/m^2^ (RSE, 1.49%) and *CL* was 5.74 L/(h•m^2^) (RSE, 1.92%), and on the q6h schedule (*n* = 174) the *V* was 16.3 L/m^2^ (RSE, 2.01%) and *CL* was 5.22 L/(h•m^2^) (RSE, 1.94%).

Busulfan *CL* was lower in patients monitored on day 2 (*n* = 167) and day 3 (*n* = 39) after dose adjustments compared to the *CL* on day 1 (Supplemental Table 5). The median decrease in *CL* from day 1 to 2 was 11%. Supplemental Fig. 5 shows the percent change in busulfan *CL*_*i*_ by dosing schedule from day 1 to day 2.

**Fig. 2:**
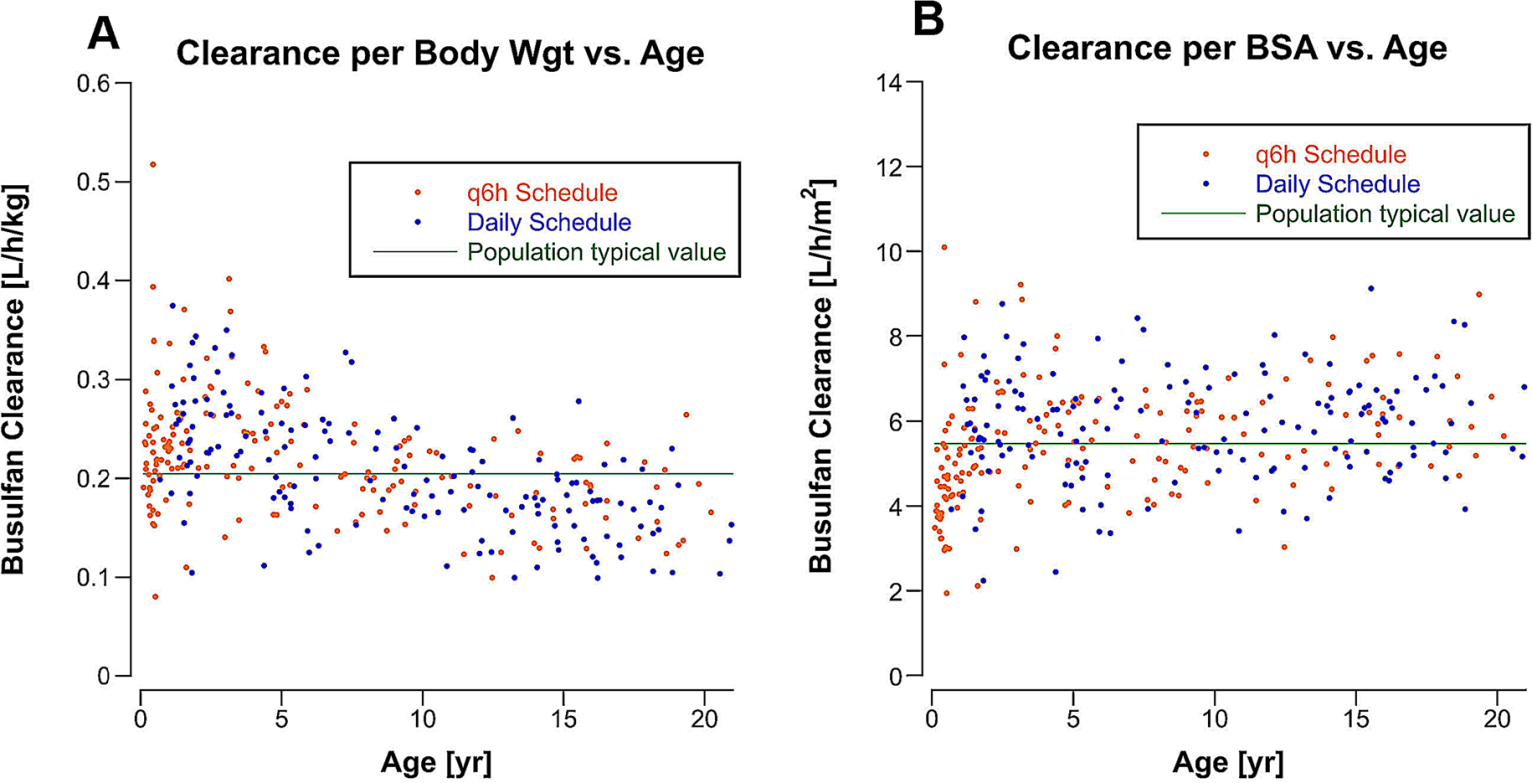
Busulfan clearance (*CL*_*i*_) scaled to body weight (**A**) and body surface area (**B**) according to patient age and by dosing schedule for the first dose of busulfan in 328 patients. The C.V. for *CL*_*i*_ normalized to BSA (L/[h•m^2^]) was 23%, normalized to body weight (L/[h•kg]) was 28%, and not normalized to body size (L/h) was 62%

Body weight and BSA were evaluated as covariates for *CL* and *V* with dose input as ng. The Stepwise Covariant Search mode selected BSA as a covariate for *CL* and *V* (Supplemental Table 6), indicating that interpatient variability is better accounted for by scaling the dose to BSA than body weight. Supplemental Fig. 6 shows the relationship between BSA and the random error term for busulfan *CL* ($\:{e}^{{\eta\:Cl}_{i}}$). Figure 2 shows individual busulfan clearances (*CL*_*i*_) scaled to body weight (L/[h•kg]) and BSA (L/[h•m^2^]) according to age. *CL*_*i*_ scaled to BSA is more uniform across the population age span than *CL*_*i*_ scaled to body weight, except in infants.

### Simulated AUC with BSA-scaled dosing

**Fig. 3:**
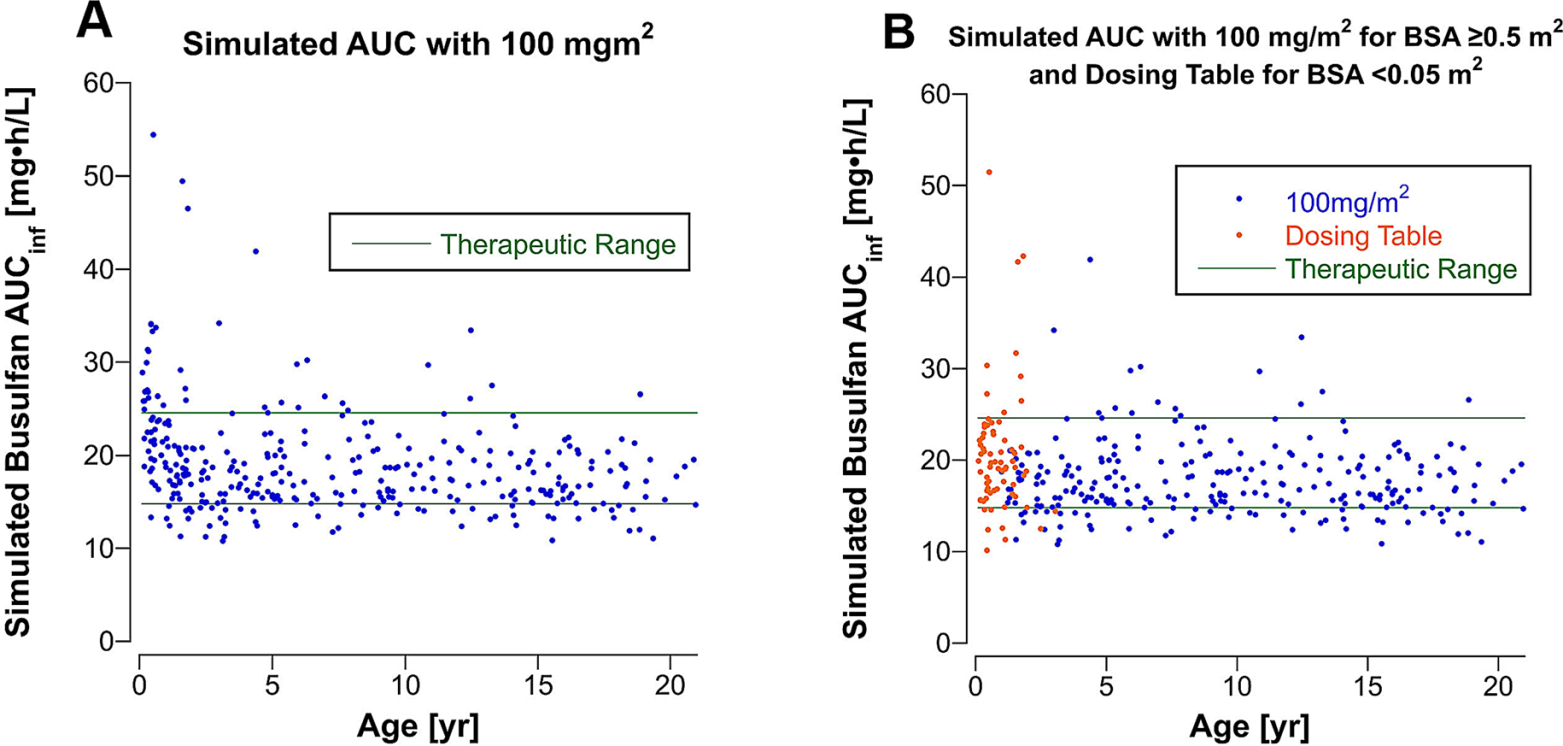
Simulated busulfan *AUC*_*inf*_ for (**A**) 328 patients using a busulfan dose of 100 mg/m^2^ and for (**B**) the 258 patients with a BSA ≥ 0.5 m^2^ using a dose of 100 mg/m^2^ plus from the BSA-banded dosing table for the 70 patients with a BSA < 0.5 m^2^. The green horizontal lines are the upper and lower bound of the therapeutic range

Simulated busulfan *AUC*_*inf*_ for doses scaled to BSA (100 mg/m^2^) and doses taken from the BSA-banded dosing table (Table 1) for patients with a BSA < 0.5 m^2^ are shown in Supplemental Table 7. A higher percentage of patients would achieve a busulfan *AUC*_*inf*_ within the therapeutic range after the first dose with BSA-scaled dosing in conjunction with the dosing table for those with a BSA < 0.5 m^2^ compared to body weight-scaled dosing. Figure 3 shows the simulated *AUC*_*inf*_ with BSA-scaled dosing (100 mg/m^2^) for all patients versus BSA-scaled dosing combined with a dose from the dosing table for patients with a BSA < 0.5 m^2^. One-third of patients with a BSA < 0.5 m^2^ are predicted to have an *AUC*_*inf*_ above the therapeutic range if they had received 100 mg/m^2^, whereas if the dosing table is used to determine the dose, only 13% would have an *AUC*_*inf*_ exceeding 24.6 (mg•h)/L on day 1. Supplemental Fig. 7 shows the percent change in the administered dose comparing the body weight-scaled dosing to the approach combining BSA-scaled dosing (BSA ≥ 0.5 m^2^) and looking up the dose in the BSA-banded dosing table (BSA < 0.5 m^2^).

Mean ± SD simulated *AUC*_*inf*_ for 100 mg/m^2^ of busulfan in 9 patients who received AIBW-scaled doses for obesity was 17.3 ± 3.5 mg•h/L, and the simulated *AUC*_*inf*_ was subtherapeutic in 2 patients (Supplemental Table 8). The patients’ actual weights were used to derive the BSA for the simulation.

## Discussion

Busulfan clearance is age-dependent when scaled to body weight in children (Fig. 2A). As a result, busulfan *AUC*_*inf*_s were subtherapeutic in 48% of children < 5 years old with a body weight-scaled dose, even with a 25% higher dose per kg in patients > 10 kg and < 48 months old. In patients > 10 years old, 78% had a *CL*_*i*_ scaled to body weight below the population *tvCL* (0.205 L/[h•kg]). Busulfan *CL*_*i*_s scaled to BSA are uniform across the pediatric age range except in infants, as observed with other anticancer drugs. Simulation of BSA scaled dosing with 100 mg/m^2^ for patients with a BSA ≥ 0.5 m^2^ and a dose from the dosing table (Table 1) for patients with a BSA < 0.5 m^2^ predicts that a substantial increase in the number of patients achieving an *AUC*_*inf*_ within the therapeutic range with the first dose (Supplemental Table 7). BSA-scaled dosing with BSA-banded dosing tables for the youngest patients is becoming the standard dosing method in COG clinical trials and, as this analysis demonstrates, could also be applied to anticancer drugs in high-dose pre-SCT conditioning regimens.

Mean busulfan *CL* scaled to body weight in 9 patients with obesity was 30% less than the *tvCL* (Supplemental Table 8), but mean *CL* scaled to BSA was < 10% different from *tvCL*. Simulation of BSA-scaled dosing (100 mg/m^2^) in these patients yielded an *AUC*_*inf*_ similar to that achieved with doses scaled to AIBW. The BSA in these patients was derived from their actual weight and height, indicating that adjustments for obesity would not be required if busulfan dose was scaled to BSA instead of body weight.

The most dramatic changes in drug elimination pathways occur during the first year of life, but pharmacokinetic data for most anticancer drugs are limited or lacking in this age group. Infants have usually been excluded from initial dose-finding studies where pharmacokinetics are performed. An added benefit of implementing TDM for busulfan has been the generation of large pharmacokinetic data sets that include infants and other special populations, and these data have facilitated refining infant dosing [13, 14]. Despite the low therapeutic indices of anticancer drugs, TDM is not widely used to individualize doses of other anticancer drugs, even though drug exposures are often highly variable. Busulfan is an exception because pharmacokinetic studies defined a therapeutic range [1], but in the absence of a defined therapeutic range, TDM could also be used to reduce inter-patient variability by adjusting doses to achieve a drug concentration or AUC within the interquartile range for the monitored variable, for example. This approach could benefit individual patients whose drug concentrations/AUCs are outliers, which could put them at risk for more severe toxicity if high or an inadequate response if low; and would provide large datasets for population pharmacokinetic studies to study covariates associated with variability and refine dosing.

We did not explore more complex covariate models of body weight because of the difficulty in translating these models into practical and safe clinical adaptive dosing methods. Poinsignon et al. developed a dosing nomogram from a complex covariate model incorporating body weight and a maturation function [13]. From the final model, they developed a dosing table that provides a busulfan dose in mg/kg for 5 body weight bands. We compared this dosing method to our proposed BSA-based dosing method in the 328 patients included in our population (Supplemental Fig. 8). The median absolute difference in the doses for the 2 dosing methods was 9.0%. The prescribed dose was higher in 87% of patients using the weight-based dosing table.

In our patients who were monitored on day 2, busulfan *CL* declined by a median of 11% from day 1. This decline in busulfan clearance over the 4-day treatment course has been previously reported [1] and has been attributed to depletion of glutathione (GSH). Conjugation of busulfan with GSH, which can occur spontaneously or can be catalyzed by glutathione-S transferases, is the first step in the busulfan metabolic pathway [15]. If GSH concentration is rate-limiting for conjugation of busulfan, then pre-treatment GSH concentration could be predictive of busulfan clearance and therefore of its *AUC*_*inf*_, and, if so, pre-treatment GSH concentration could potentially be incorporated into adaptive dosing methods for busulfan.

In our population, which spanned the pediatric age range, simulated BSA-scaled dosing (100 mg/m^2^) with modified doses for the youngest patients using a dosing table appears to result in more patients achieving an *AUC*_*inf*_ within the therapeutic range after the first dose, including in patients with obesity. Traditionally, populations are divided into test and validation groups for dosing simulations, but we elected not to split our population so that clearances from all of the youngest patients could be used to develop the dosing table. Instead, we plan to perform a pilot clinical study to validate this dosing method prospectively.

## Electronic supplementary material

Below is the link to the electronic supplementary material.

Supplementary Material 1

Supplementary Material 2

## Data Availability

No datasets were generated or analysed during the current study.
