# Supplementary material for: New approach to busulfan dosing in infants and children based on a population pharmacokinetic analysis
Source: Cancer Chemother Pharmacol. 2025 Feb 11;95(1):32. doi: 10.1007/s00280-025-04757-w (PMC11813957; doi:10.1007/s00280-025-04757-w)

Supplemental Table 1

Data elements abstracted from each patient's medical record.

**Data Elements**
Date of birth to calculate precise age on day of monitoring
Sex
Weight, height, and body surface area on the day prior to first busulfan dose
Diagnosis
Other anticancer drugs in transplant preparative regimen
Donor relationship
Pre-treatment laboratory tests<br>Creatinine<br>Albumin<br>Bilirubin<br>Alanine transaminase
Diagnosis of sinusoidal obstructive syndrome post-BMT
Diagnosis of graft rejection post-BMT
Busulfan dose information<br>Dose in mg for every busulfan dose<br>Dosing interval (daily or q6h)<br>Date of every busulfan dose<br>Start and end times of every busulfan infusion<br>Date and time each busulfan plasma sample was drawn

Supplemental Table 2

Nominal plasma sampling times for busulfan timed from the start of the busulfan infusion.

	**Nominal Plasma Sampling Times [h]**			
	**Daily schedule**		**q6h schedule**	
**Sample #**	**1st dose**	**2nd, 3rd dose**	**1st dose**	**2nd, 3rd dose**
1	3 (EOI*)	Pre-dose	2 (EOI)	Pre-dose
2	3.25	3 (EOI)	2.25	2 (EOI)
3	3.5	3.25	2.5	2.25
4	4	3.5	3	2.5
5	5	5	4	4
6	6	7	5	6
7	8		6	

* EOI, End of infusion

Supplemental Table 3

Data sets and dose units analyzed by fitting a one-compartment model using a population pharmacokinetic approach. Gray cells indicate inclusion in the analysis and each row represents a separate analysis.

<table>
<tr><th colspan="2">Schedule</th><th colspan="3">Day of Monitoring</th><th colspan="3">Dose Units</th><th>Age Group</th><th>Covariates</th><th>n</th></tr>
<tr><th>Daily</th><th>q6h</th><th>1st</th><th>2nd</th><th>3rd</th><th>ng</th><th>ng/kg</th><th>ng/m2</th><th>[yrs]</th><th></th><th></th></tr>
<tr><td bgcolor="#d9d9d9"></td><td bgcolor="#d9d9d9"></td><td bgcolor="#d9d9d9"></td><td></td><td></td><td bgcolor="#d9d9d9"></td><td></td><td></td><td>All</td><td>-</td><td>328</td></tr>
<tr><td bgcolor="#d9d9d9"></td><td bgcolor="#d9d9d9"></td><td bgcolor="#d9d9d9"></td><td></td><td></td><td></td><td bgcolor="#d9d9d9"></td><td></td><td>All</td><td>-</td><td>328</td></tr>
<tr><td bgcolor="#d9d9d9"></td><td bgcolor="#d9d9d9"></td><td bgcolor="#d9d9d9"></td><td></td><td></td><td></td><td></td><td bgcolor="#d9d9d9"></td><td>All</td><td>-</td><td>328</td></tr>
<tr><td bgcolor="#d9d9d9"></td><td bgcolor="#d9d9d9"></td><td bgcolor="#d9d9d9"></td><td></td><td></td><td bgcolor="#d9d9d9"></td><td></td><td></td><td>All</td><td>Wgt, BSA</td><td>328</td></tr>
<tr><td bgcolor="#d9d9d9"></td><td></td><td bgcolor="#d9d9d9"></td><td></td><td></td><td></td><td></td><td bgcolor="#d9d9d9"></td><td>All</td><td>-</td><td>154</td></tr>
<tr><td></td><td bgcolor="#d9d9d9"></td><td bgcolor="#d9d9d9"></td><td></td><td></td><td></td><td></td><td bgcolor="#d9d9d9"></td><td>All</td><td>-</td><td>174</td></tr>
<tr><td bgcolor="#d9d9d9"></td><td bgcolor="#d9d9d9"></td><td bgcolor="#d9d9d9"></td><td></td><td></td><td bgcolor="#d9d9d9"></td><td></td><td></td><td><3</td><td>-</td><td>108</td></tr>
<tr><td bgcolor="#d9d9d9"></td><td bgcolor="#d9d9d9"></td><td bgcolor="#d9d9d9"></td><td></td><td></td><td></td><td bgcolor="#d9d9d9"></td><td></td><td><3</td><td>-</td><td>108</td></tr>
<tr><td bgcolor="#d9d9d9"></td><td bgcolor="#d9d9d9"></td><td bgcolor="#d9d9d9"></td><td></td><td></td><td></td><td></td><td bgcolor="#d9d9d9"></td><td><3</td><td>-</td><td>108</td></tr>
<tr><td bgcolor="#d9d9d9"></td><td bgcolor="#d9d9d9"></td><td bgcolor="#d9d9d9"></td><td></td><td></td><td bgcolor="#d9d9d9"></td><td></td><td></td><td><2</td><td>-</td><td>88</td></tr>
<tr><td bgcolor="#d9d9d9"></td><td bgcolor="#d9d9d9"></td><td bgcolor="#d9d9d9"></td><td></td><td></td><td></td><td bgcolor="#d9d9d9"></td><td></td><td><2</td><td>-</td><td>88</td></tr>
<tr><td bgcolor="#d9d9d9"></td><td bgcolor="#d9d9d9"></td><td bgcolor="#d9d9d9"></td><td></td><td></td><td></td><td></td><td bgcolor="#d9d9d9"></td><td><2</td><td>-</td><td>88</td></tr>
<tr><td bgcolor="#d9d9d9"></td><td bgcolor="#d9d9d9"></td><td bgcolor="#d9d9d9"></td><td></td><td></td><td bgcolor="#d9d9d9"></td><td></td><td></td><td><1</td><td>-</td><td>46</td></tr>
<tr><td bgcolor="#d9d9d9"></td><td bgcolor="#d9d9d9"></td><td bgcolor="#d9d9d9"></td><td></td><td></td><td></td><td bgcolor="#d9d9d9"></td><td></td><td><1</td><td>-</td><td>46</td></tr>
<tr><td bgcolor="#d9d9d9"></td><td bgcolor="#d9d9d9"></td><td bgcolor="#d9d9d9"></td><td></td><td></td><td></td><td></td><td bgcolor="#d9d9d9"></td><td><1</td><td>-</td><td>46</td></tr>
<tr><td bgcolor="#d9d9d9"></td><td bgcolor="#d9d9d9"></td><td></td><td bgcolor="#d9d9d9"></td><td></td><td></td><td></td><td bgcolor="#d9d9d9"></td><td>All</td><td>-</td><td>167</td></tr>
<tr><td bgcolor="#d9d9d9"></td><td bgcolor="#d9d9d9"></td><td bgcolor="#d9d9d9">*</td><td></td><td></td><td></td><td></td><td bgcolor="#d9d9d9"></td><td>All</td><td>-</td><td>167</td></tr>
<tr><td bgcolor="#d9d9d9"></td><td bgcolor="#d9d9d9"></td><td></td><td></td><td bgcolor="#d9d9d9"></td><td></td><td></td><td bgcolor="#d9d9d9"></td><td>All</td><td>-</td><td>39</td></tr>
<tr><td bgcolor="#d9d9d9"></td><td bgcolor="#d9d9d9"></td><td bgcolor="#d9d9d9">**</td><td></td><td></td><td></td><td></td><td bgcolor="#d9d9d9"></td><td>All</td><td>-</td><td>39</td></tr>
<tr><td bgcolor="#d9d9d9"></td><td bgcolor="#d9d9d9"></td><td></td><td bgcolor="#d9d9d9">***</td><td></td><td></td><td></td><td bgcolor="#d9d9d9"></td><td>All</td><td>-</td><td>39</td></tr>
</table>

* Includes data for the day 1 monitoring only from the 167 subjects who were monitored on day 2

** Includes data for the day 1 monitoring only from the 39 subjects who were monitored on day 3

*** Includes data for the day 2 monitoring only from the 39 subjects who were monitored on day 3

Supplemental Table 4

Other drugs administered with busulfan in conditioning regimen.

**Conditioning Regimen with Busulfan**	**N**
ATG*, fludarabine	55
ATG, fludarabine, thiotepa	54
Cyclophosphamide, thiotepa	35
Melphalan	35
ATG, cyclophosphamide, fludarabine	32
Cyclophosphamide	31
ATG, cyclophosphamide, thiotepa	30
ATG, cyclophosphamide	21
None	8
Fludarabine	7
Fludarabine, melphalan	7
ATG, fludarabine, melphalan	5
ATG	2
ATG, cyclophosphamide, melphalan	2
Cyclophosphamide, fludarabine	1
Cyclophosphamide, fludarabine, thiotepa	1
Cyclophosphamide, melphalan	1
Fludarabine, thiotepa	1

* ATG, anti-thymocyte globulin

Supplemental Table 5

Typical values for busulfan *CL* on days 2 and 3. The day 1 *CL* values are matched and include only the patients who were monitored on day 2 or 3.

**Day of Dose**	**N**	***CL* (RSE) [L/(h•m^2)]**
Patients monitored on day 2		
Day 2	167	5.13 (2.20)
Day 1	167	5.76 (2.01)
Patients monitored on day 3		
Day 3	39	4.91 (8.05)
Day 2	39	4.76 (6.23)
Day 1	39	5.64 (5.52)

Supplemental Table 6

Model parameters for 1-compartment pharmacokinetic model incorporating BSA as a covariate for model parameters *V* and *CL*. Bootstrap analysis included 1,000 samples with a maximum of 10 tries, and 98.2% of the samples converged. The objective function (-2•log likelihood) for the base model without BSA as a covariant was 28025 and with BSA as a covariant for *V* and *CL* was 26380. For the final model with BSA as a covariant for *CL* and *V*, the variance from the Omega matrix for ηCL is 0.0572 and for ηV is 0.0279. For the 1-compartment base model without BSA as a covariant and dose input in ng, the variance for ηCL is 0.473 and for ηV is 0.595.

			**Bootstrap Analysis**	
**Parameter**	**Estimate**	**RSE [%]**	**Estimate**	**RES [%]**
tvV [L]	14.4	0.91	14.3	7.08
tvCl [L/h]	4.42	1.33	4.41	6.07
dVdBSA	1.33	1.46	1.34	1.49
dCLdBSA	1.14	2.20	1.14	2.22

Supplemental Table 7

Simulated busulfan AUC_{inf} for a 100 mg/m^2 dose of bulsufan and for the BSA-banded infant dosing table (Table 1) for patients with a BSA <0.5 m^2. 100 mg/m^2 was selected based on the 30-rule for converting dose per kg to dose to m^2 (3.2 mg/kg • 30 kg/m^2 = 96 mg/m^2). 30 is the weight to BSA ratio at 10 years of age. Dose per kg is the actual AUC_{inf} achieved after a dose scaled to body weight.

	**Number (%) of Patients within each AUC_{inf} Range**			
**Dosing**	**14.8-24.6* mg•h/L**	**<14.8 mg•h/L**	**>24.6 mg•h/L**	**Median AUC [mg•h/L]**
All Patients (n=328)				
Dose per kg	166 (50.6)	136 (41.5)	24 (7.3)	15.8
100 mg/m^2	234 (71.3)	56 (17.1)	38 (11.6)	18.0
100 mg/m^2 BSA ≥0.5 m^2 Dosing table BSA <0.5 m^2	247 (75.3)	57 (17.4)	24 (7.3)	17.6
Patients with BSA <0.5 m^2 (n=70)				
Dose per kg	36 (51.4)	31 (44.3)	3 (4.3)	15.3
100 mg/m^2	40 (57.1)	7 (10.0)	23 (32.9)	21.7
Dosing table	53 (75.7)	8 (11.4)	9 (12.9)	19.5

* Therapeutic range; 14.8 (mg•h)/L = 3600 μM•min and 24.6 (mg•h)/L = 6000 μM•min.

Supplemental Table 8

Results for 9 patients who received busulfan dosed based on AIBW (adjusted ideal body weight) due to obesity. The AUC_{inf} was simulated for a dose of 100 mg/m^2 using the BSA derived from each patient's actual weight and height.

				**Daily Dose**			**Clearance**			
**Age [yr]**	**Wgt [kg]**	**AIBW [kg]**	**BSA [m^2]**	**[mg]**	**[mg/kg]**	**[mg/m^2]**	**L/(h•kg)**	**L/(h•m^2)**	**Actual AUC$_{inf}$ [mg•h/L]**	**Sim AUC$_{inf}$ [mg•h/L]†**
11.5	41.4	34.4	1.24	112	2.71	90.3	0.123	4.13	22.3	24.2
9.4	75.1	65	1.78	208	2.77	117	0.154	6.44	17.7	15.5
16.2	112	83/57*	2.28	183	1.63	80.3	0.099	4.79	16.5*	20.9
12.0	45.1	36	1.31	115	2.55	87.8	0.192	6.57	12.8	15.2
20.5	126	85.1	2.42	273	2.17	113	0.104	5.35	21.4	18.7
20.9	94.3	74	2.11	237	2.51	112	0.153	6.80	16.1	14.7
15.5	116	87.3	2.36	280	2.41	118	0.139	6.73	17.0	14.9
14.8	83.6	66	1.92	211	2.52	110	0.128	5.52	20.1	18.1
9.7	67.4	48.3	1.67	154	2.28	92.2	0.184	7.26	12.0	13.8
				**Mean**	2.40	102	0.142	5.95	17.3	17.3
				**SD**	0.34	15	0.032	1.05	3.6	3.5
			**Population *CL***				0.205	5.47		

* Patient's calculated AIBW was 83 kg, but actual dose was scaled to 57 kg

† Simulated AUC$_{inf}$ for a dose of 100 mg/m^2 using each patient's actual BSA

Supplemental Figure Legends

Supplemental Figure 1. Histogram of the age distribution of the 328 patients included in this study.

Supplemental Figure 2. Percent change in the busulfan AUC_{inf} from day 1 to day 2 as a function of the percent change in the dose from day 1 to day 2 in 156 patients.

Supplemental Figure 3. Predicted plasma busulfan (BU) concentrations from the one-compartment population pharmacokinetic model with covariate (BSA) vs. the measured plasma concentrations after the first dose of busulfan in 328 patients (2149 concentrations). **A.** predicted concentrations using the population parameters (*tvV* and *tvCl*); **B.** predicted concentrations using each patient's individual parameters (V_i and CL_i). **C.** Conditional weighted residuals as a function of Time after the start of the busulfan infusion.

Supplemental Figure 4. Busulfan clearance after the first dose by sex. Median CL_i in females was 5.38 L/(h•m^2) and median CL_i in males was 5.65 L/(h•m^2).

Supplemental Figure 5. Percent change in busulfan CL_i from day 1 to day 2 by dosing schedule (daily or q6h) in 167 patients who were monitored after the first dose on day 2. Percent change in clearance = ((CL_i day 2 – CL_i day 1)/CL_i day 1)•100.

Supplemental Figure 6. Relationship between the BSA of individual patients and their random error term for busulfan clearance ($e^{\eta Cl_i}$) from the one-compartment model fit to the busulfan concentration-time data after the first dose (n=328) with dose input as ng without **(A)** and with **(B)** BSA as a covariate. Based on a linear regression analysis on Fig 4A, BSA accounts for 86% of the variability in $e^{\eta Cl_i}$.

Supplemental Figure 7. Percent change in busulfan dose for the 328 patients for a dose of 100 mg/m^2 for patients with a BSA ≥0.5 m^2 or for a dose taken from the BSA-banded dosing table for patients with a BSA <0.5 m^2 relative to their dose scaled to their body weight. Patients who

were >10 kg and <48 months received a 25% higher dose per kg, so their BSA-scaled dose does not result in as large of a percent increase.

Percent change in dose = ((100 mg/m^2 • BSA_i) – (3.2 or 4 mg/kg • Wgt_i)/(3.2 or 4 mg/kg • Wgt_i))•100 OR Percent change in dose = (dose from BSA-banded dosing table – (3.2 or 4 mg/kg • Wgt_i)/(3.2 or 4 mg/kg • Wgt_i))•100.

Supplemental Figure 8. Comparison of the BSA-scaled busulfan dosing method presented in this study to a previously published body weight-scaled dosing table method.[13] Doses were calculated for the 328 patients included in our population using their body weights and BSAs and the percent difference in the doses prescribed by the 2 dosing methods was calculated for each patient using this formula: ((dose from the body weight-scaled dosing table – dose from the BSA-scaled dosing method)/dose from the body weight-scaled dosing table)*100. A positive percentage difference means the dose from the body weight-scaled method is higher.

Supplemental Figure 1. Histogram of the age distribution of the 328 patients included in this study.

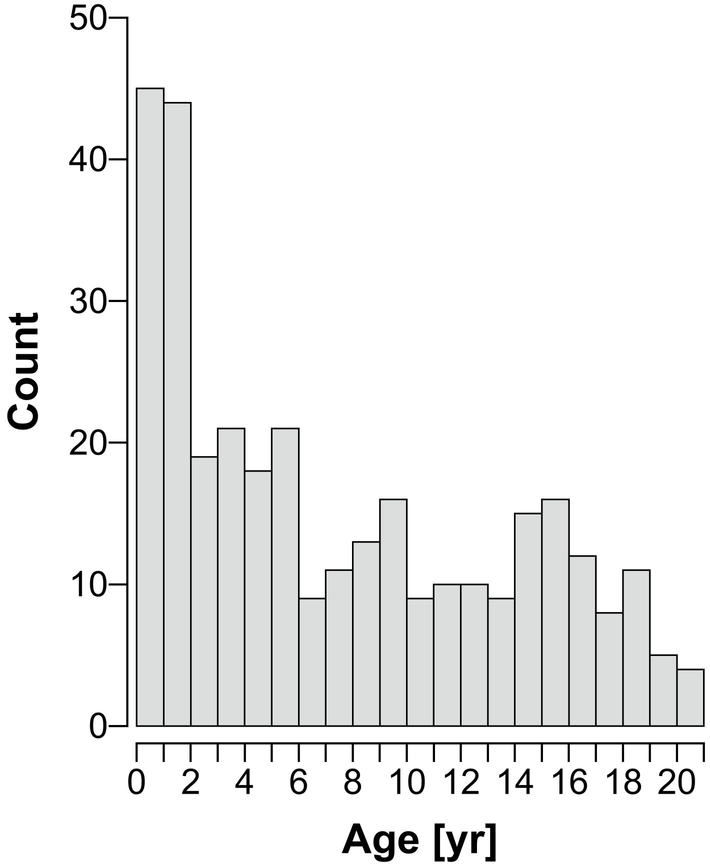

Supplemental Figure 2. Percent change in the busulfan AUC_{inf} from day 1 to day 2 as a function of the percent change in the dose from day 1 to day 2 in 156 patients.

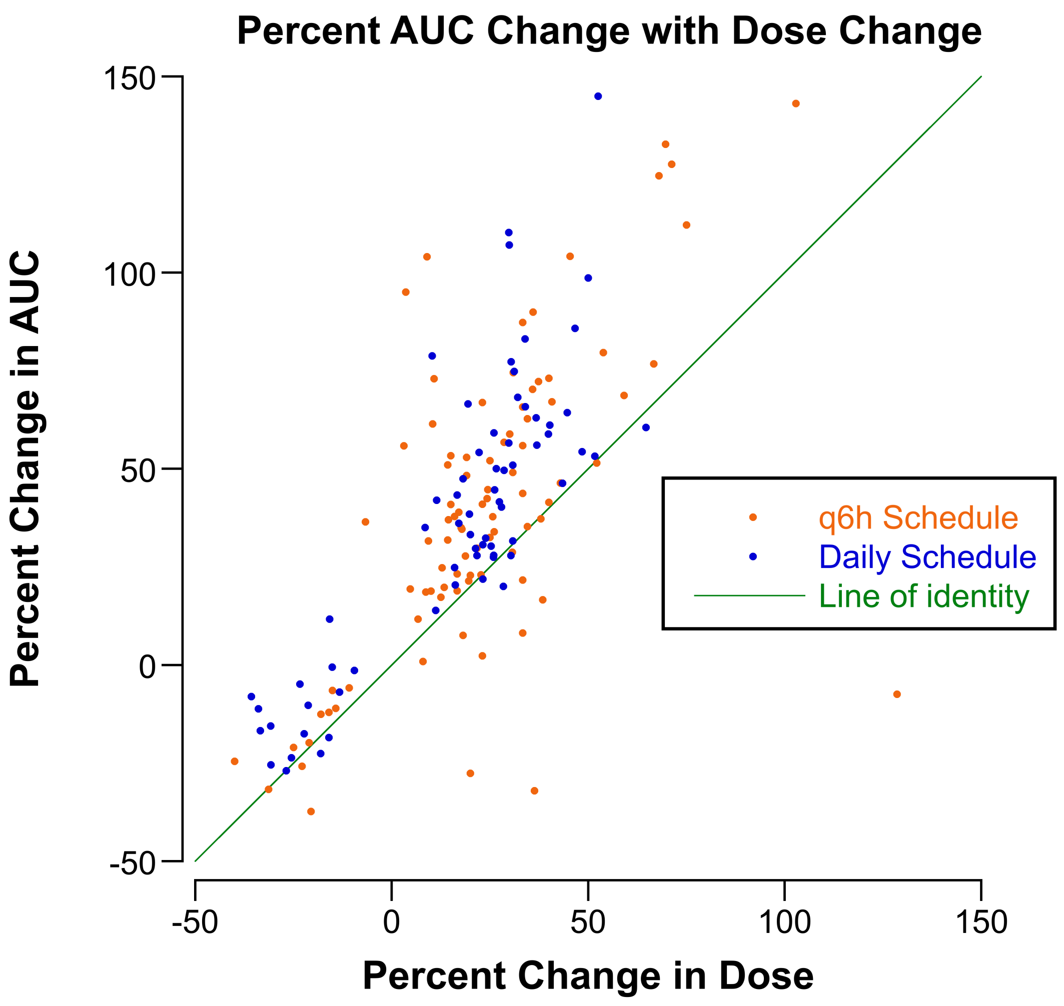

Supplemental Figure 3. Predicted plasma busulfan (BU) concentrations from the one-compartment population pharmacokinetic model with covariate (BSA) vs. the measured plasma concentrations after the first dose of busulfan in 328 patients (2149 concentrations). **A.** predicted concentrations using the population parameters (*tvV* and *tvCl*); **B.** predicted concentrations using each patient's individual parameters (V_i and CL_i). **C.** Conditional weighted residuals as a function of Time after the start of the busulfan infusion.

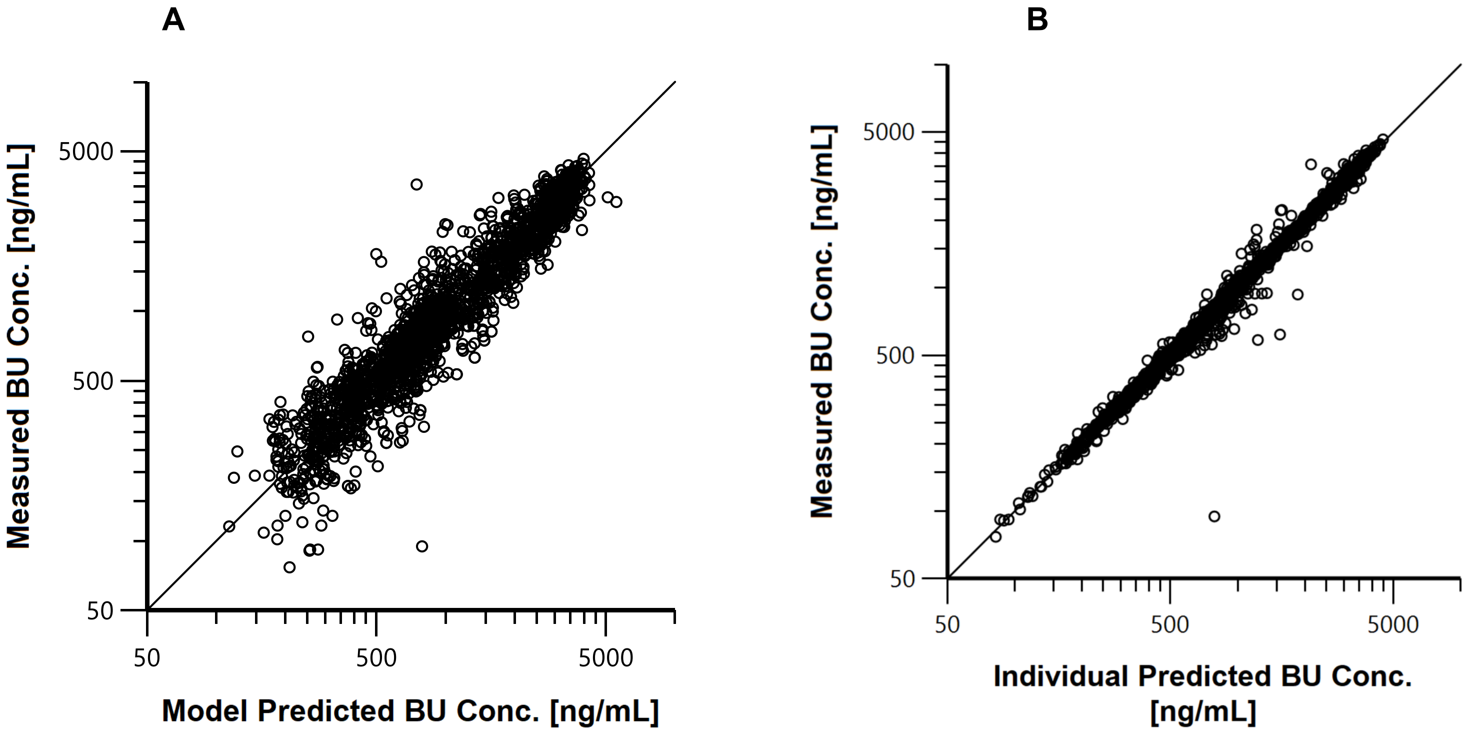

C

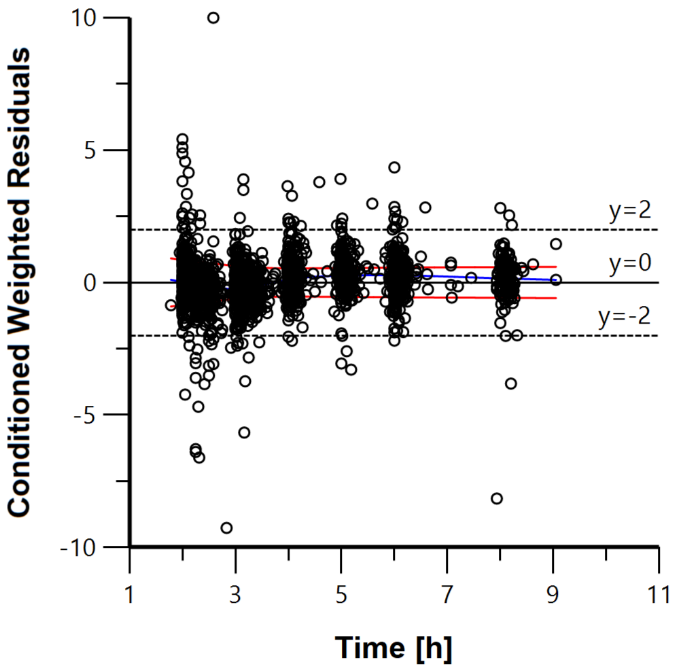
Conditioned Weighted Residuals
10
5
0
-5
-10
1
3
5
7
9
11
Time [h]
y=2
y=0
y=-2

Supplemental Figure 4. Busulfan CL_i after the first dose by sex. Median CL_i in females was 5.38 L/(h•m^2) and median CL_i in males was 5.65 L/(h•m^2).

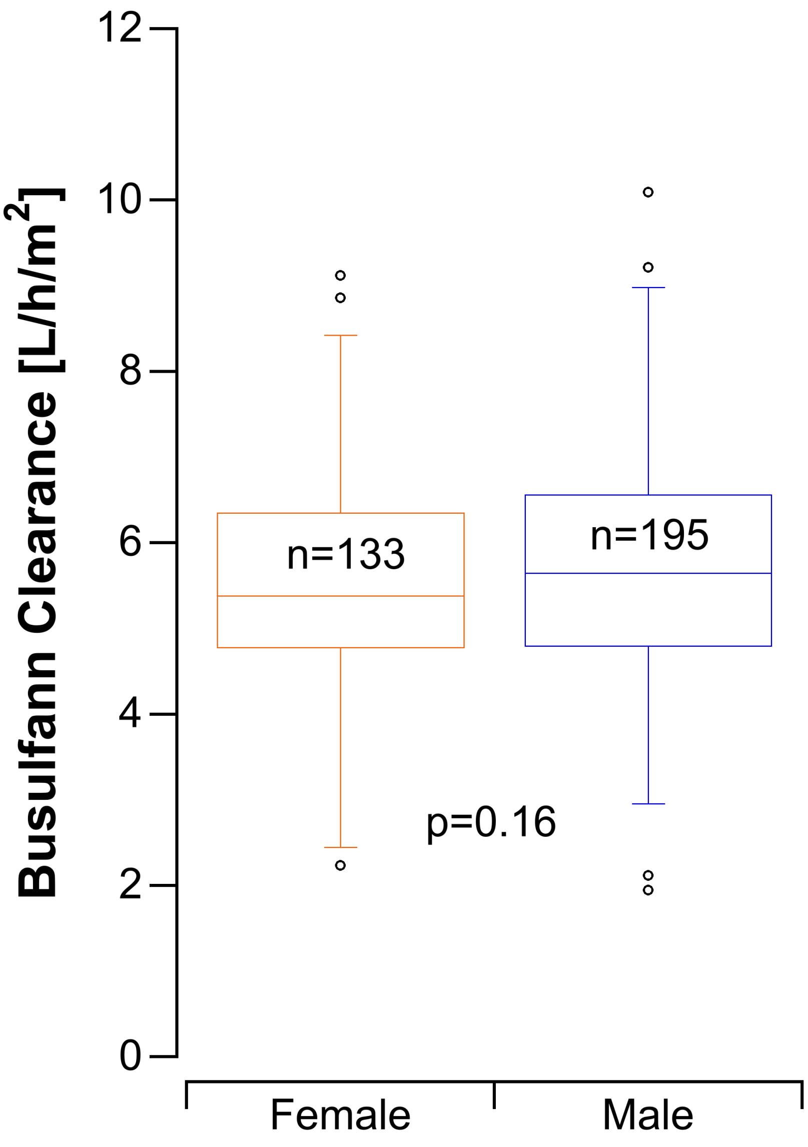

Supplemental Figure 5. Percent change in busulfan CL_i from day 1 to day 2 by dosing schedule (daily or q6h) in 167 patients who were monitored after the first dose on day 2. Percent change in clearance = ((CL_i day 2 – CL_i day 1)/CL_i day 1)•100.

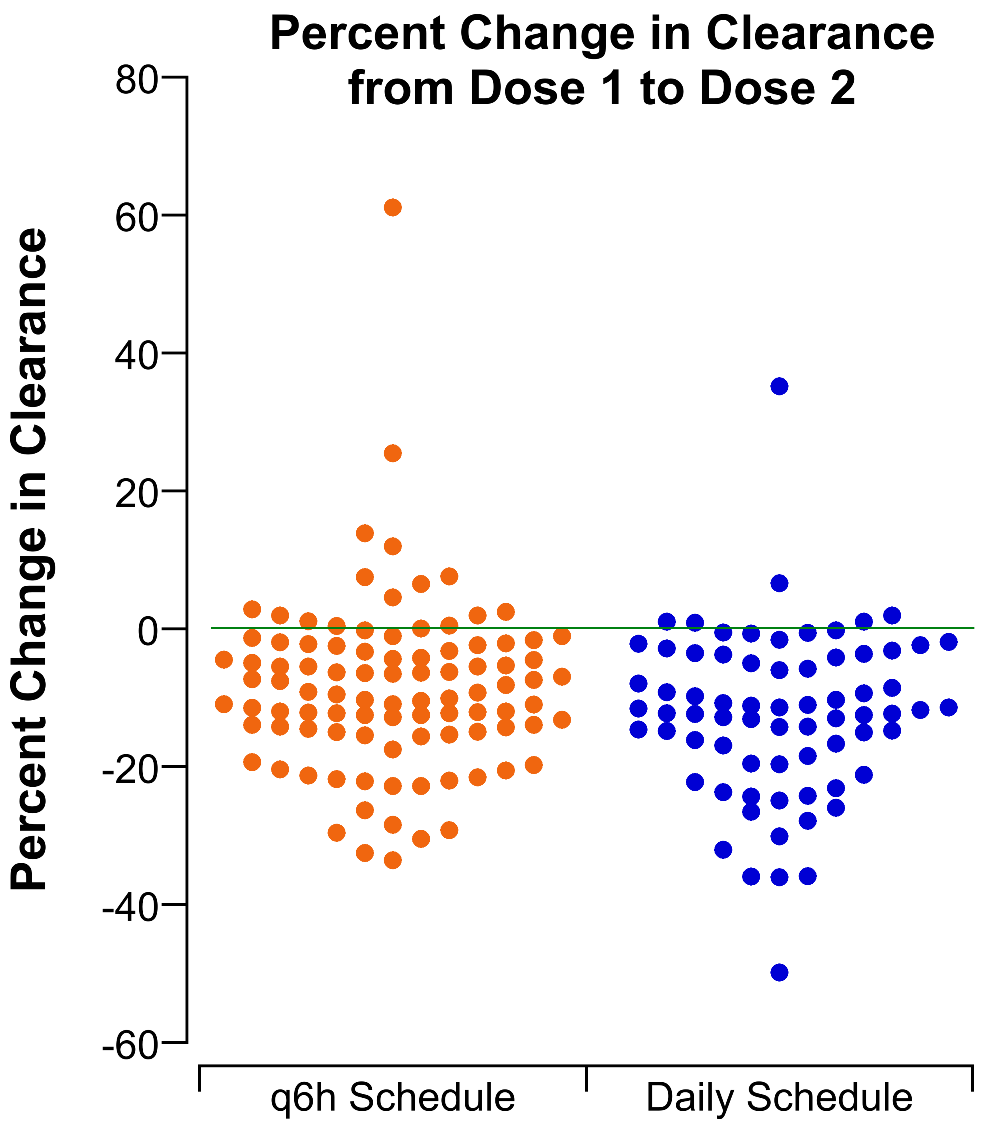

Supplemental Figure 6. Relationship between the BSA of individual patients and their random error term for busulfan clearance ($e^{\eta Cl_i}$) from the one-compartment model fit to the busulfan concentration-time data after the first dose (n=328) with dose input as ng without **(A)** and with **(B)** BSA as a covariate. Based on a linear regression analysis on Fig 4A, BSA accounts for 86% of the variability in $e^{\eta Cl_i}$.

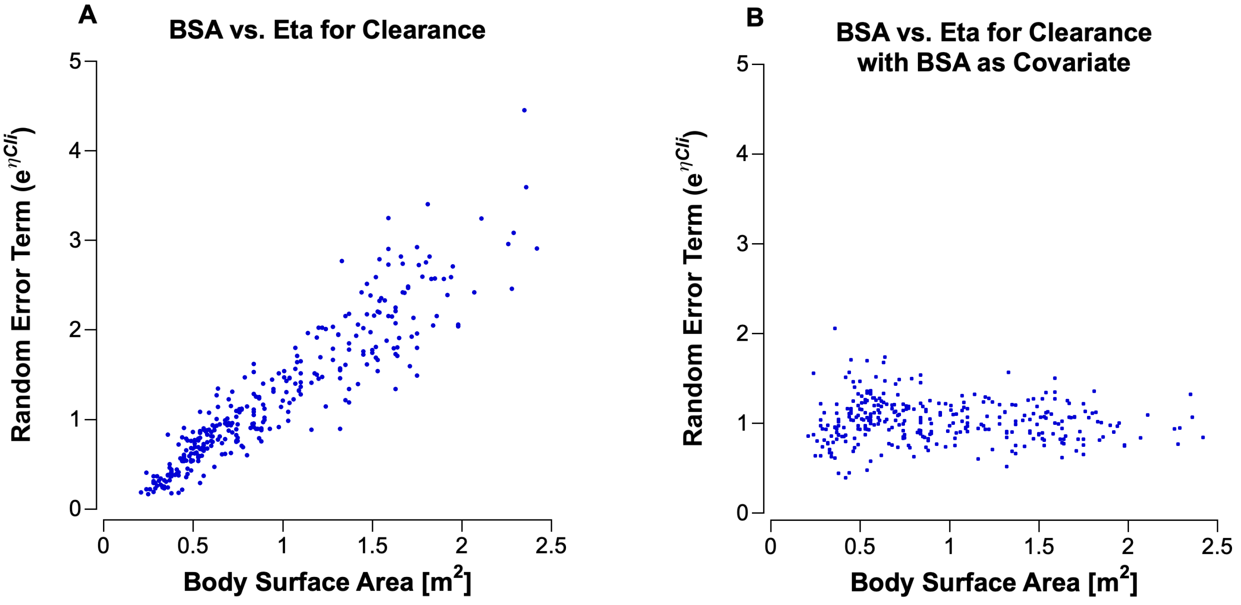

Supplemental Figure 7. Percent change in busulfan dose for the 328 patients for a dose of 100 mg/m^2 for patients with a BSA ≥0.5 m^2 or for a dose taken from the BSA-banded dosing table for patients with a BSA <0.5 m^2 relative to their dose scaled to their body weight. Patients who were >10 kg and <48 months received a 25% higher dose per kg, so their BSA-scaled dose does not result in as large of a percent increase.

Percent change in dose = ((100 mg/m^2 • BSA_i) – (3.2 or 4 mg/kg • Wgt_i)/(3.2 or 4 mg/kg • Wgt_i))•100 OR Percent change in dose = (dose from BSA-banded dosing table – (3.2 or 4 mg/kg • Wgt_i)/(3.2 or 4 mg/kg • Wgt_i))•100.

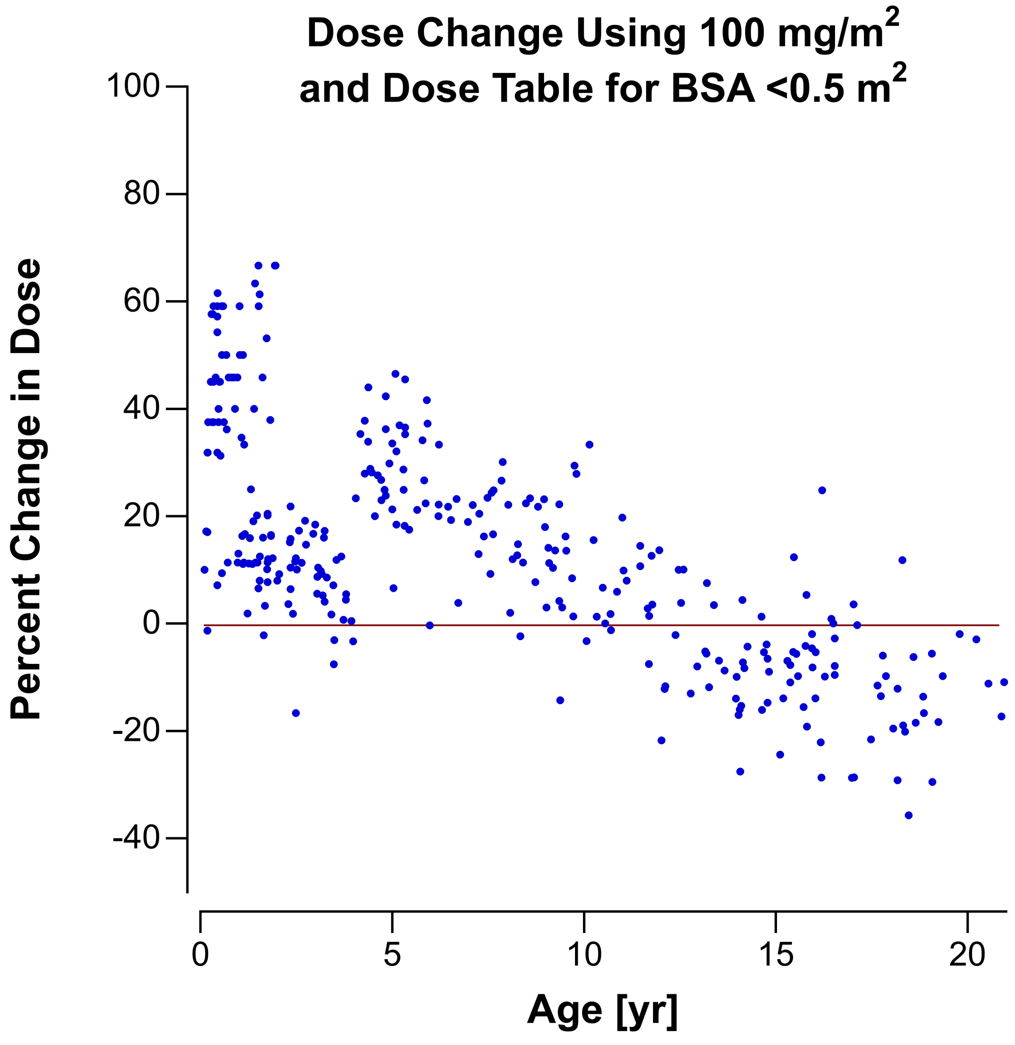

Supplemental Figure 8. Comparison of the BSA-scaled busulfan dosing method presented in this study to a previously published body weight-scaled dosing table method.[13] Doses were calculated for the 328 patients included in our population using their body weights and BSAs and the percent difference in the doses prescribed by the 2 dosing methods was calculated for each patient using this formula: ((dose from the body weight-scaled dosing table – dose from the BSA-scaled dosing method)/dose from the body weight-scaled dosing table)*100. A positive percentage difference means the dose from the body weight-scaled method is higher.

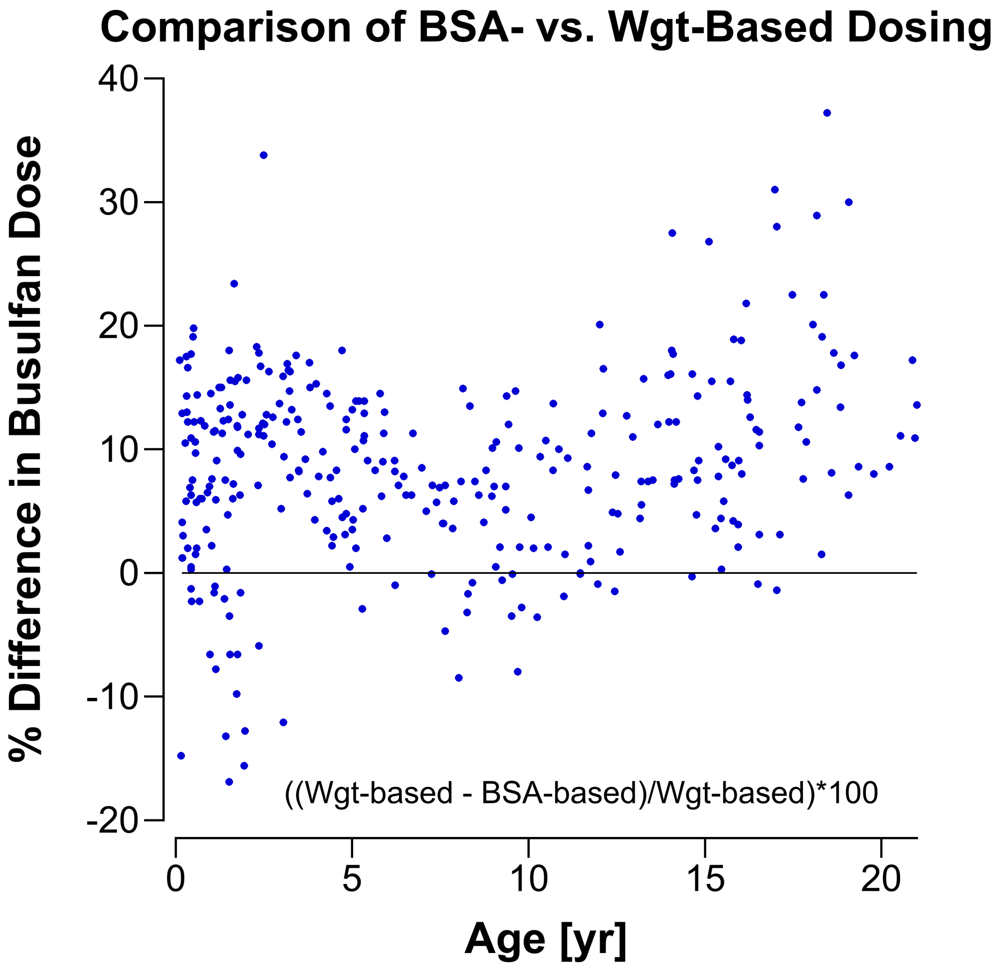

Supplement: Supplementary file 2 — Supplementary Material 2 [file 280_2025_4757_MOESM2_ESM.pdf]
